# Casualty Officers

**Published:** 1954

**Authors:** 


### CASUALTY OFFICERS

Some hospitals appear to be having difficulty in recruiting casualty officers. One hospital secretary goes so far as to say that it will not be possible fully to staff a hospital casualty department with newly qualified men in the future, and that some new system will have to be thought out. The position at Cossham Hospital in Bristol became so serious that in recent months the Secretary has had to circularize the general practitioners inviting them to help in the casualty de ment on a sessional basis. Sometimes good comes of necessity, and this m the way that the general practitioners are going to get a foot in the hosp We understand there has not been too ready a response from general practitio moreover those who have undertaken the work are complaining that some doctors seem to have a habit of sending to the casualty department much work which they should be doing themselves.

Developments will be watched with interest.

